# Supplementary figures and images for: A novel ferroptosis-related signature for predicting prognosis, immune characteristics, and treatment prediction in hepatocellular carcinoma
Source: PLoS One. 2025 Jun 4;20(6):e0322158. doi: 10.1371/journal.pone.0322158 (PMC12136417; doi:10.1371/journal.pone.0322158)

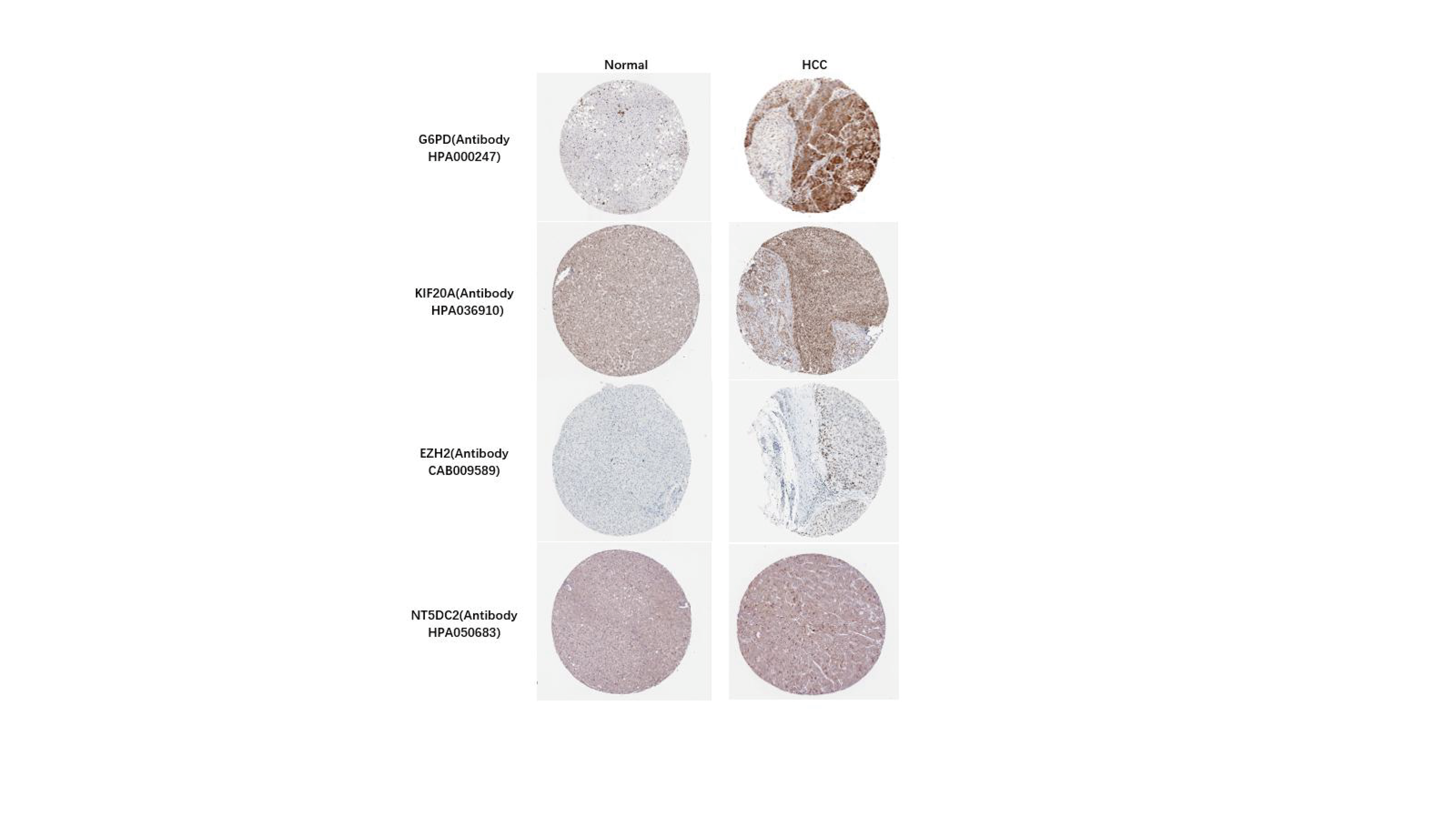

Supplement: S1 Fig — (TIF) [file pone.0322158.s001.tif]

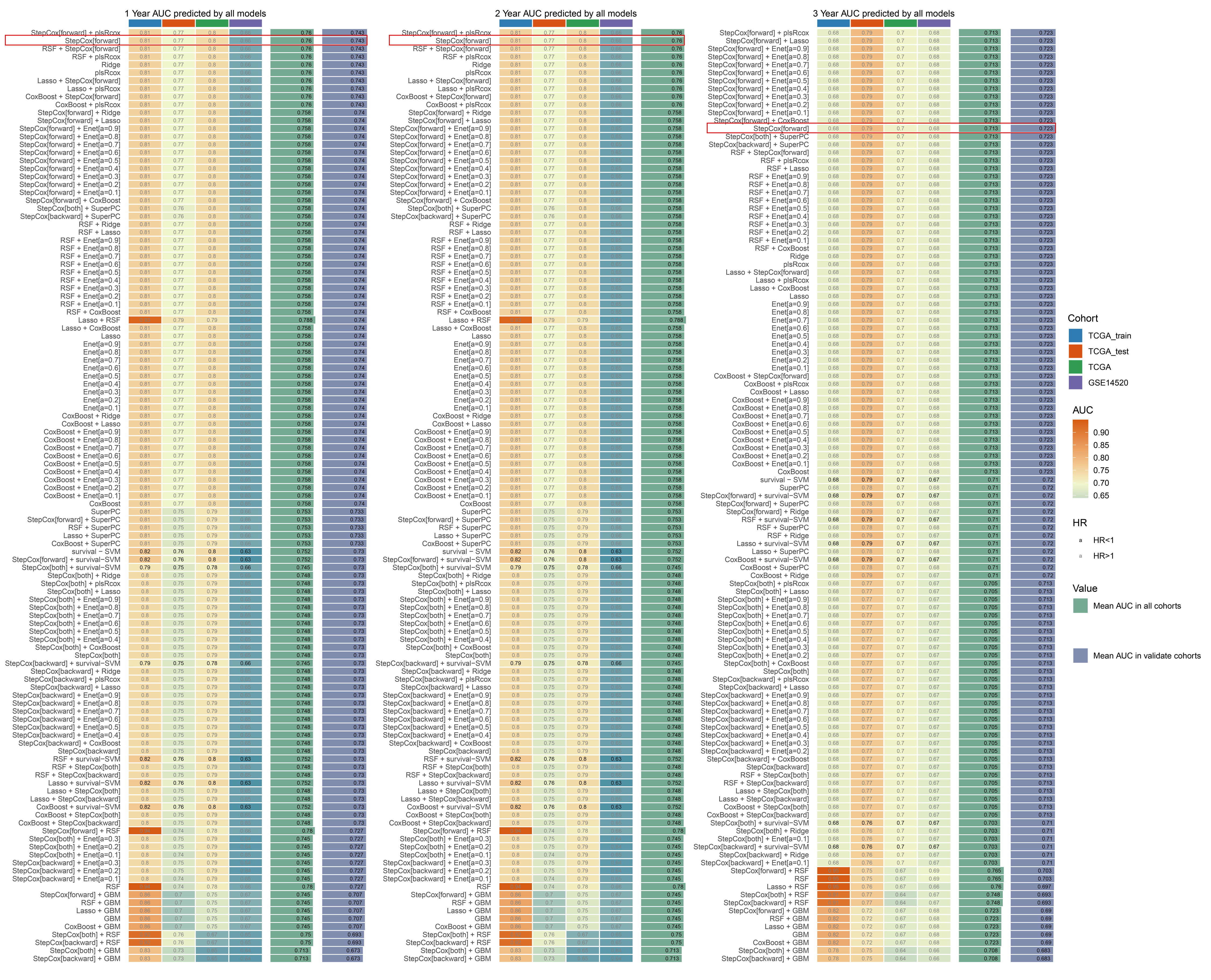

Supplement: S2 Fig — (TIF) [file pone.0322158.s002.tif]

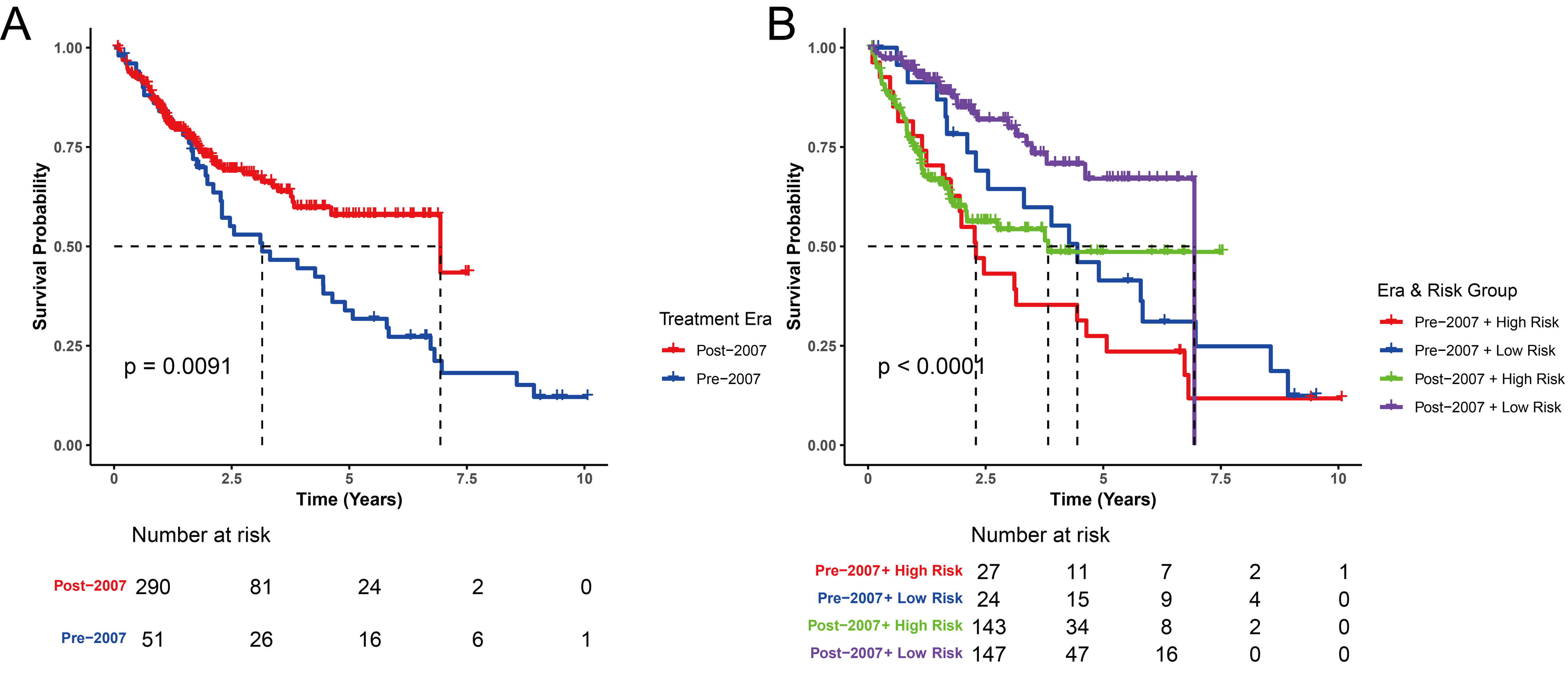

Supplement: S3 Fig — (TIF) [file pone.0322158.s003.tif]

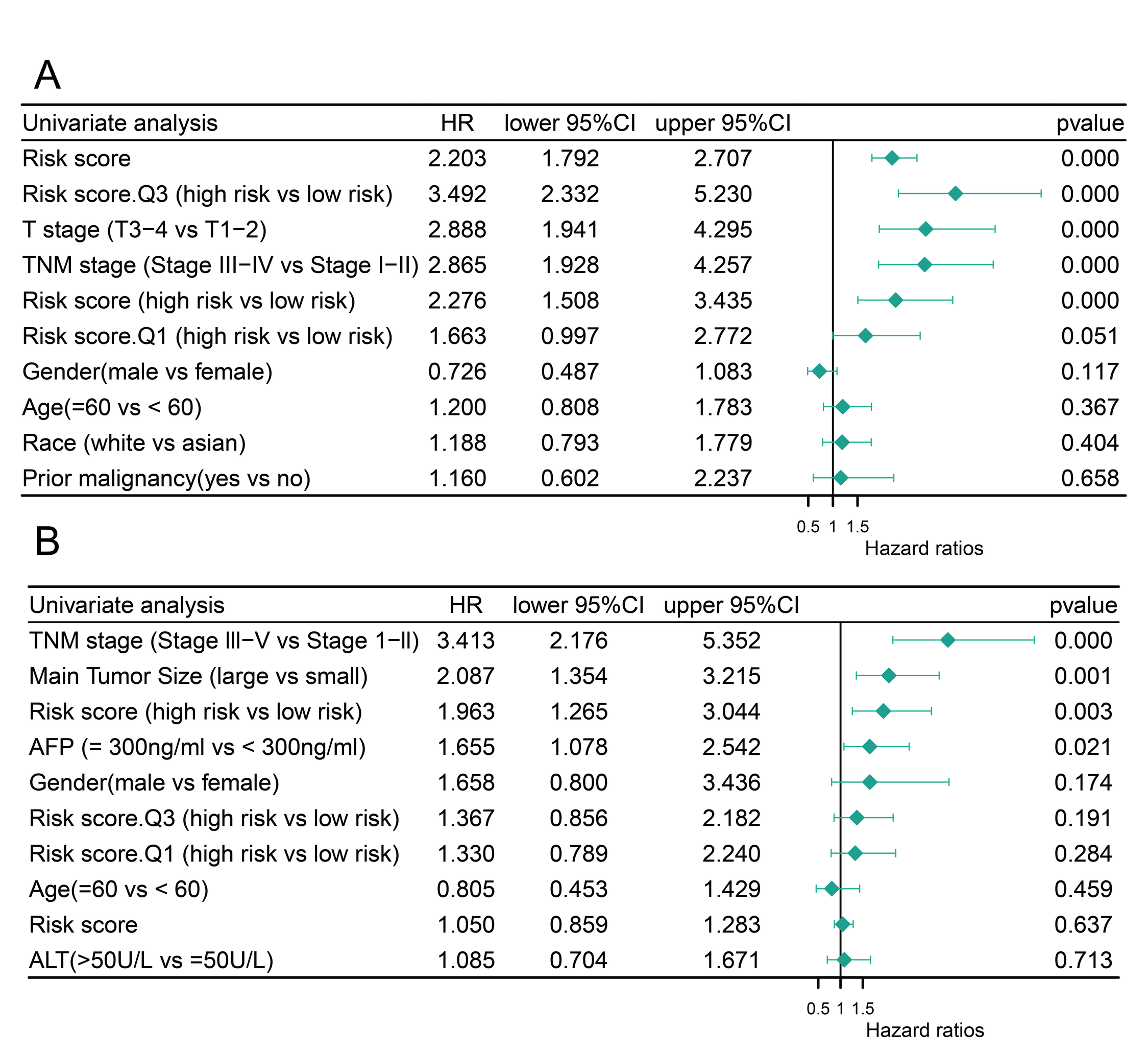

Supplement: S4 Fig — (TIF) [file pone.0322158.s004.tif]

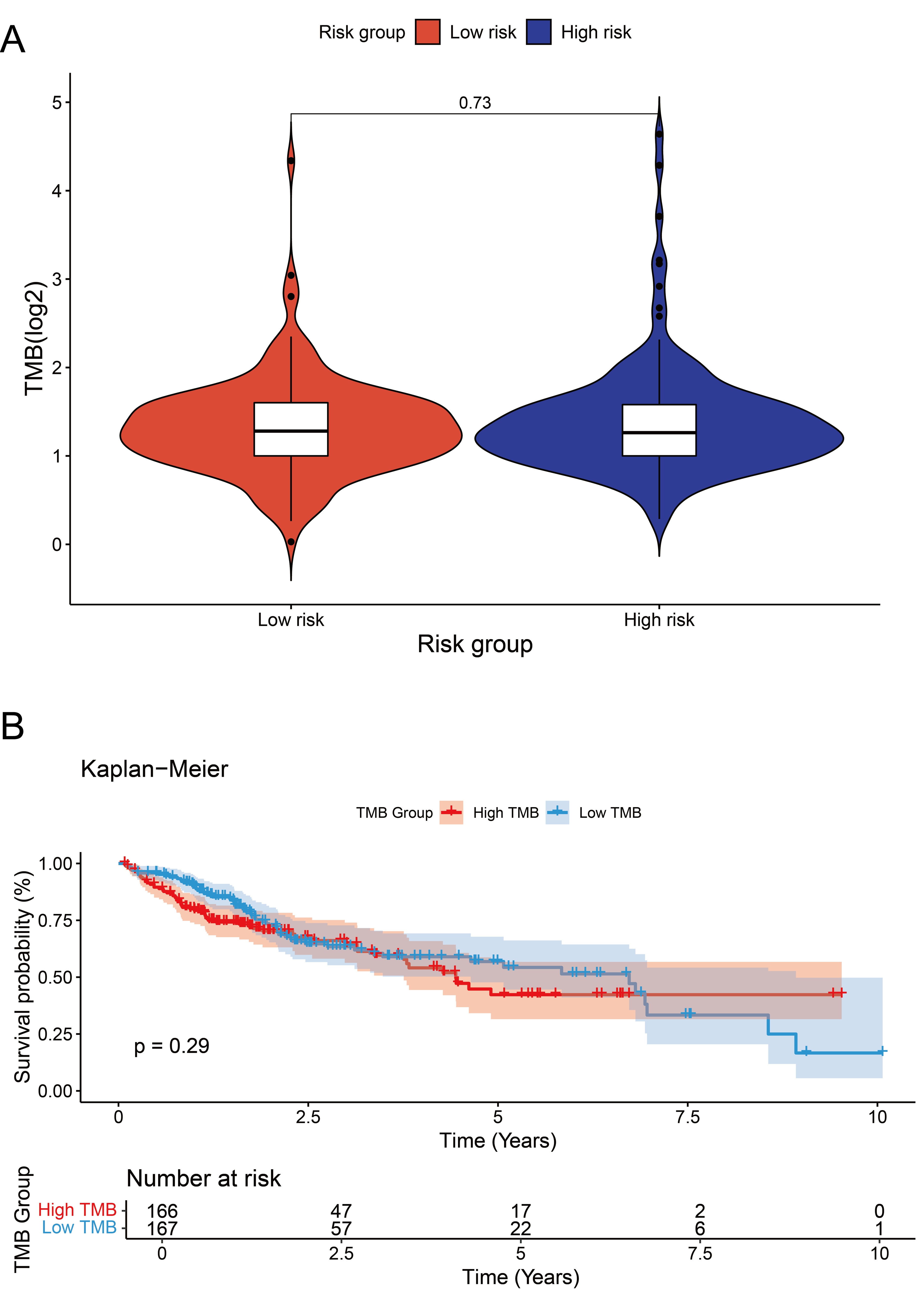

Supplement: S6 Fig — (TIF) [file pone.0322158.s006.tif]

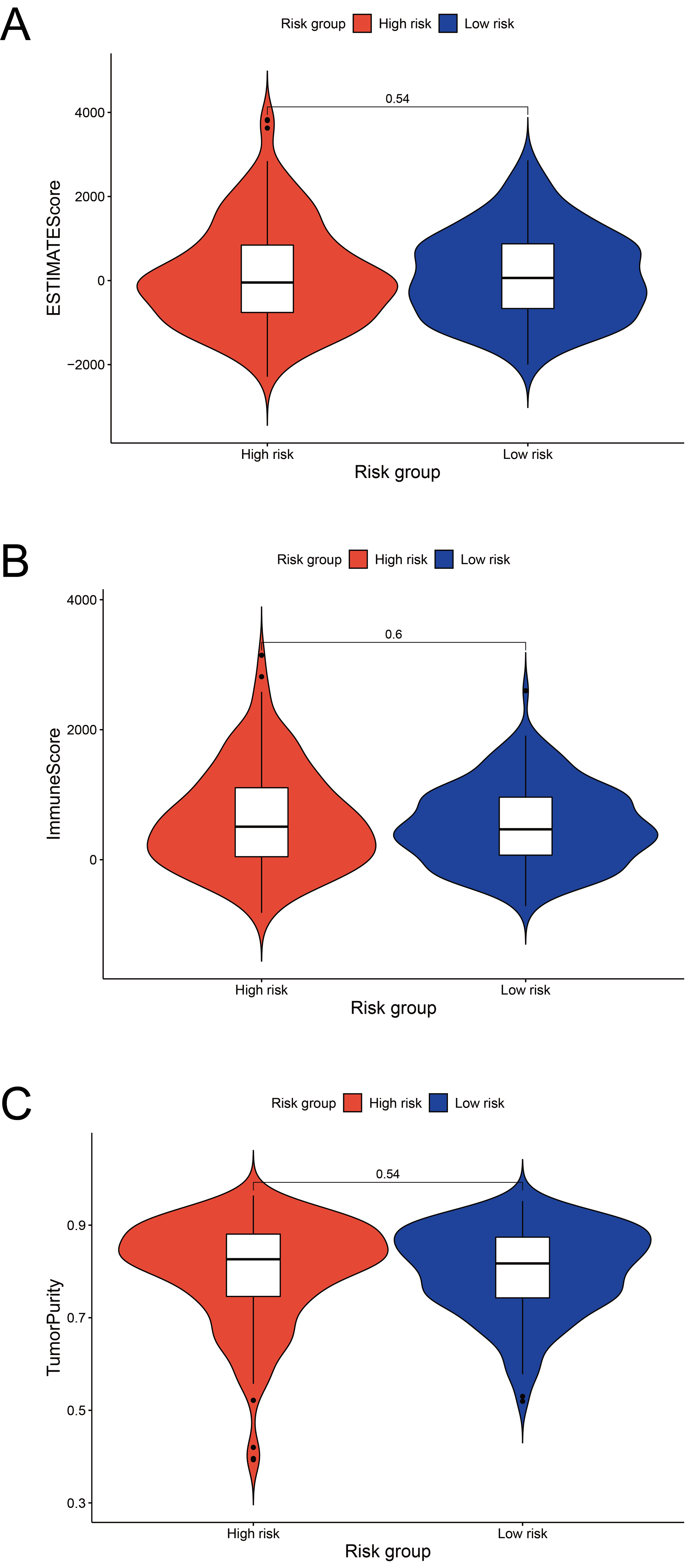

Supplement: S7 Fig — (TIF) [file pone.0322158.s007.tif]
