# Supplementary material for: A novel ferroptosis-related signature for predicting prognosis, immune characteristics, and treatment prediction in hepatocellular carcinoma
Source: PLoS One. 2025 Jun 4;20(6):e0322158. doi: 10.1371/journal.pone.0322158 (PMC12136417; doi:10.1371/journal.pone.0322158)
Supplement: Attachment — Submitted filename: Response to Reviewers.docx [file pone.0322158.s014.docx]

**Reviewer #1:**

**Main Issues:**

**1、Inconsistent p-value formatting**

We have carefully reviewed all p-values in the manuscript and ensured consistent formatting. All p-values are now uniformly presented as follows: p < 0.05 indicates significance, and p < 0.01 indicates high significance. The specific changes are as follows:

Line 214: Changed to “p < 0.05”.

Line 329: Changed to "** for p < 0.01, * for p < 0.05".

Line 332: Changed to “p < 0.01”.

Lines 439 and 441: Formatting has been unified and corrected.

Additionally, changes have been made to lines 140, 213, 347, 363, and 437 to ensure p < 0.05 is used consistently.

**2、Grammar and formatting errors in the text**

We have thoroughly reviewed and corrected the grammatical and formatting issues in the manuscript.

Lines 266, 466, 467, 468, and 662 have been revised to address grammar and formatting issues.

**3、Condensing the description of Figures 8, 9, 10, and 12**

We have simplified the description of Figure 12 to make it more concise and clear, removing redundant information.

**4、Resolution issues with Figures 8, 9, 10, and 12**

We have remade these figures and increased their resolution to ensure clarity when zoomed in.

**5、Addition of Figure 1, including immunohistochemistry images**

We have added a scale bar and annotation markers to Figure 1 to enhance its readability and informativeness.

**Reviewer #2:**

**Below is our detailed response to each of your comments:**

**1、Regarding the missing information on patient RNA sample sources and clinical features:**

We appreciate your reminder, and we agree that additional information on patient demographics and clinical features is necessary. In the revised manuscript, we have added the demographic characteristics (including age, sex, etc.) and relevant clinical features of patients from the TCGA and GSE14520 datasets. These additions are included in the supplementary materials and referenced in the main text on pages 352 and 363. The supplementary tables are Table S3 and Table S4.

**2、Regarding your suggestion about using the median as a cutoff value:**

Thank you for your valuable suggestion. We understand your concern that using the median as a cutoff may be too simplistic and may not fully reflect biological or clinical differences. After careful consideration, we have decided to retain the use of median-based grouping. We believe this method is simple yet effective in categorizing patients into high-risk and low-risk groups, and it has shown satisfactory prognostic stratification in our study. Additionally, median-based grouping is widely used in biomedical research and has clinical practicality. However, we acknowledge that using more granular groupings might offer more detailed risk stratification. If possible, we will mention this point in the discussion and explore finer risk stratification methods in future studies. Once again, we appreciate your insightful suggestion, and we believe the current grouping method still supports the results of our model effectively. We welcome any further suggestions you may have.

**3、Regarding inconsistent bracket formatting:**

Thank you very much for pointing out this issue. We have reviewed the manuscript and corrected all instances of non-standard Chinese brackets, replacing them with standard English brackets to ensure consistent formatting.

**4、Regarding the discussion on the five genes and their mechanisms in ferroptosis:**

Your feedback is greatly appreciated. We have expanded the discussion in the revised manuscript to include a detailed exploration of the potential mechanisms between the five genes and the regulation of ferroptosis pathways. Specifically, we discuss the interactions of these genes in ferroptosis and their potential roles in hepatocellular carcinoma (HCC), which has been added to the discussion section.

**5、Regarding the assumptions of the statistical methods:**

In the revised version, we have provided a more detailed discussion of the assumptions underlying the statistical methods used in our study. We specifically point out that the univariate and multivariate Cox regression models used in our study are based on the proportional hazards assumption, which may not hold in all cases. We further discuss the impact of any violations of this assumption on the interpretation of results and emphasize the importance of model validation. Additionally, we have included a discussion of the limitations associated with LASSO regression, particularly regarding the potential omission of some promising genes during variable selection.

We have also expanded the discussion of the model's limitations, especially its application in different clinical contexts. Although the model performed well in both the training and testing datasets, the potential bias in dataset selection and sample heterogeneity means that external validation and generalization in real-world settings still require further verification. Therefore, we suggest that future research should focus on enhancing the model’s stability and generalizability through methods such as cross-validation and validation using external datasets.

**Reviewer #3:**

Thank you for your valuable feedback on our manuscript. We have carefully reviewed the issues raised and made corresponding revisions and improvements. Below is our detailed response to each issue:

**Major Issues:**

**1、Low Image Quality:**

We appreciate your feedback regarding the image quality. We have re-made all the figures (except Figures 1, 2, and 7) and improved the resolution to ensure that all images are clear when zoomed in. The updated high-resolution images have been incorporated into the manuscript for your reference.

**2、Contradictions in Results and Logical Gaps:**

Thank you for pointing out this contradiction. Regarding the prognostic contradiction related to the high-risk group and their potential benefit from immune checkpoint inhibitors (ICIs), we have provided a detailed explanation in the revised manuscript. We acknowledge that although the high-risk group has a poorer prognosis and may exhibit immune evasion and immune suppression characteristics within the tumor microenvironment (TME), they may have a reduced response to ICIs. In contrast, the low-risk group, with weaker immune suppression and stronger immune responsiveness, may benefit more from ICIs. We have expanded the discussion section to explore this point in more detail and provide potential mechanisms that explain the difference in response to ICIs between the risk groups.

**3、Insufficient Explanation of Terms:**

We have added brief definitions for the TIDE and ESTIMATE scores and their relevance to hepatocellular carcinoma (HCC) in the manuscript. We have explained how these scores assist in evaluating immune evasion, tumor immune microenvironment, and their relationship with treatment response. This additional content has been included in the “Methods” section, and we further clarified the significance and role of these terms in the discussion section.

**4、Missing Background on Datasets:**

We appreciate your comment on the data background. We confirm that the TCGA and GSE14520 datasets do not provide specific treatment information for the patients. Therefore, our study primarily focuses on clinical features, gene expression data, and relevant biomarkers to perform prognostic analysis and risk stratification.

For the "prognosis" related statements, we validated the model's prognostic ability through various statistical methods, including Cox regression analysis and Kaplan-Meier survival curve analysis. We also performed validation in an independent dataset, demonstrating the model's stability and applicability in different clinical contexts. Despite the lack of treatment information, these analyses remain of significant clinical importance.

Regarding the "prediction" part, our method explicitly assesses the potential response to immunotherapy based on the patients' genetic characteristics and immune microenvironment. For example, the TIDE score and immune evasion analysis provide an initial prediction of the patient's response to ICIs. Nevertheless, we emphasize that due to the absence of specific treatment data, the clinical application value of these prediction results needs further validation and exploration.

**5、Lack of Experimental Validation:**

Thank you for highlighting the issue of experimental validation. We fully understand that experimental validation is crucial for enhancing the credibility and clinical relevance of the study. However, the focus of this study is primarily on bioinformatics analysis based on TCGA and other public datasets. Experimental validation requires significant resources and time investment, and our current research budget and time constraints limit us from conducting in vitro or in vivo experiments at this stage.

Nevertheless, we believe that the results based on multidimensional data analysis and cross-validation provide reliable support for the potential application of the nFRGs model in HCC. We also recognize that experimental validation is a key step in advancing the research further, and we plan to focus on clinical sample validation and functional experiments in future research to strengthen the clinical relevance of this model.

We have added a detailed discussion of this limitation in the revised manuscript and proposed possible directions for future experimental validation. Once again, we thank the reviewer for bringing up this important issue and for their valuable suggestions.

**Minor Issues:**

**References:**

Thank you for your comments on the references. We understand that the abstract typically does not include specific citations, and hence we did not provide references for the background information in the abstract. However, we have now included relevant references in the introduction, where we discuss the role of ferroptosis in hepatocellular carcinoma (HCC), citing relevant studies. In the revised manuscript, we will ensure that all pertinent references are accurately cited in the main text to support the arguments raised in both the abstract and introduction.

**Figures Presentation:**

Thank you for your feedback on the presentation of the figures. We greatly value the clarity and readability of the figures and have optimized them based on your suggestions. In the revised version, we have increased the size of the figures to ensure better visibility and to prevent the data from being overcrowded or hard to interpret. We believe these improvements will enhance the readability of the figures and help readers better understand our findings. Thank you for your valuable input on this matter.
