# Supplementary material for: A novel ferroptosis-related signature for predicting prognosis, immune characteristics, and treatment prediction in hepatocellular carcinoma
Source: PLoS One. 2025 Jun 4;20(6):e0322158. doi: 10.1371/journal.pone.0322158 (PMC12136417; doi:10.1371/journal.pone.0322158)
Supplement: Attachment — Submitted filename: Response_to_Reviewers_auresp_2.docx [file pone.0322158.s015.docx]

**Dear Editors and Reviewers：**

Thank you for your review and suggestions on our manuscript titled "A Novel Ferroptosis-Related Signature for Predicting Prognosis, Immune Characteristics, and Treatment Prediction in Hepatocellular Carcinoma." Based on your feedback, we have revised the manuscript and provided detailed responses to each reviewer’s comments in the sections below. Regarding the baseline characteristics matching information:

As per the reviewer’s request, we have added baseline characteristic matching tables for the two cohorts in the revised manuscript. We also conducted a comparison of key variables and included the patients' pathological staging (TNM) for comparison. The tables highlight the significant differences between the groups.

We have made all the necessary revisions as described above and improved the manuscript according to the reviewers' suggestions. Once again, we appreciate your guidance and valuable feedback on our work. We look forward to your further review and hope that this revision meets the requirements of your journal.

**Reviewer #3:**

**Main Issues:**

**1. Figures require better formatting, font size adjustments, and improved layout for readability.**

**Response:** Thank you for your valuable feedback on the formatting and readability of the figures. Based on your suggestions, we have optimized several figures, with specific changes as follows:

(1) Font size adjustment: We have increased the font size in Figures 3B-F, 4, 5A, D, E, H, 6B, and 11A to improve readability, ensuring that all legends, axis labels, and text are clearly visible.

(2) Clarification of legends and statistical methods: In Figures 3 and 4, we have clarified the meanings of "high" and "low" in the legends and added descriptions of the statistical methods used (e.g., log-rank test for Kaplan-Meier survival curve analysis, Cox regression analysis). These modifications enhance the transparency and comprehensibility of the figures.

(3) Layout optimization of Figure 6B: For Figure 6B, we have rearranged the layout, reducing large blank areas and increasing the font size to enhance both clarity and overall layout efficiency. These adjustments make the figure more compact and easier to understand.

(4) Optimization of x-axis labels in Figure 11A: We have increased the font size of the x-axis labels in Figure 11A to improve visibility and readability. We believe these improvements will significantly enhance the visual effectiveness of the figures and better convey the research findings.

Thank you again for your valuable comments, and we look forward to your further feedback.

**2.The manuscript provides only a superficial description of the data and lacks in-depth analysis. The data analysis should be deepened to explore biological mechanisms, immune microenvironment interactions, and validation in independent cohorts.**

**Response:** Thank you for your valuable comments. We take your point regarding the "superficial description of the data" seriously and are committed to further improving our analysis. In the revised manuscript, we have conducted a deeper analysis, particularly in the following areas:

(1) In-depth discussion of biological mechanisms: We have provided a more detailed exploration of how ferroptosis-related genes (G6PD, KIF20A, EZH2, NT5DC2, SLC7A11) interact to affect the tumor microenvironment (TME). We emphasize how these genes not only function in their respective signaling pathways but also regulate important biological processes, such as iron homeostasis, oxidative stress, and antioxidant defense, thus influencing the occurrence of ferroptosis. We further discuss how these genes, by regulating cellular oxidative stress balance and immune escape mechanisms, play dual roles in tumor progression, either supporting or inhibiting ferroptosis and interacting with immune responses.

(2) Interactions within the immune microenvironment: We have expanded our analysis of the immune microenvironment, particularly focusing on how these ferroptosis-related genes regulate immune cell functions to modulate immune escape or enhance immune responses. We discuss how these genes interact with immune cells to affect tumor cells' immune escape mechanisms and immune recognition processes, providing potential targets for cancer immunotherapy. These detailed analyses help us understand the complex relationships between ferroptosis, tumor progression, and immune modulation.

(3) Independent cohort validation: In response to your comment on the "lack of independent cohort validation," we have further explained the external validation using the GEO database. The GEO dataset (such as GSE14520) serves as an external validation set, providing important support for the accuracy and reproducibility of our model. To further enhance the clinical translation of our model, we also plan to incorporate independent cohort data from different geographic regions and clinical centers in future research to further validate the stability and universality of our model.

We greatly appreciate your reminder on these key issues and believe these additional analyses will effectively strengthen the depth and clinical relevance of the manuscript. If you have any further suggestions or questions, we would be happy to make further revisions and improvements.

**3. Risk stratification should go beyond median cutoff, and statistical assumptions need to be explicitly tested and justified.**

**Response:** Thank you for your valuable comment. Indeed, to better understand the clinical outcomes of different patient groups, risk stratification should go beyond a single median cutoff. Therefore, we considered multiple cutoffs in our analysis, including the median, first quartile (Q1), and third quartile (Q3), and conducted corresponding univariate Cox regression analyses. In the TCGA dataset, we found that using Q3 as a cutoff yielded the most significant effect and showed strong correlation with clinical outcomes. This suggests that the Q3 cutoff provides finer risk stratification and may reflect characteristics of certain patient groups in the dataset. However, we also recognize that differences between datasets may exist, so we re-analyzed the GSE14520 dataset. In the GSE14520 dataset, we found that using Q1 and Q3 as cutoffs did not show significant differences in clinical outcomes, while the median cutoff still provided significant risk stratification. Therefore, we concluded that the median cutoff is the most effective in this dataset. We speculate that this may be due to differences in patient characteristics and clinical outcomes between the GSE14520 and TCGA datasets. Relevant content has been added to the "Risk Model Evaluation" section of the manuscript and included in the supplementary file as Fig S4.

We fully understand that selecting the most appropriate cutoff is critical to the robustness and clinical significance of the model. To further enhance the reliability and clinical value of our model, we will continue to strengthen the statistical analysis of different cutoffs and update the results.

**4. The immune profile analysis requires better correlation with established immune scoring methods (e.g., TIS/Pembro score) to provide a more biologically meaningful interpretation.**

**Response:** Thank you for your valuable suggestion! Based on your feedback, we have further analyzed the results and included a visualization of the IPS score in Figure 13. Additionally, we have added descriptions in the Results and Discussion sections to further confirm the advantage of the low-risk group in immune therapy. Here are the updates:

(1) Update to Figure 13: In Figure 13, we have added a visualization of the IPS score, showing the immune score differences between the low-risk and high-risk groups. Through this visualization, we can more intuitively demonstrate the potential of the low-risk group in immune therapy, particularly in the CTLA4-negative and PD1-negative, as well as CTLA4-positive and PD1-negative groups, where the immune scores of the low-risk group are significantly higher than those of the high-risk group.

(2) Updates to Results and Discussion sections: In the Results and Discussion sections, we further describe the predictive roles of TIDE and IPS scores in immune therapy. We found that the low-risk group had a lower TIDE score, indicating a lower degree of immune escape, which may make them more responsive to immune therapy. Additionally, the IPS score of the low-risk group was significantly higher than that of the high-risk group, further supporting the advantage of the low-risk group in immune therapy. The combined analysis of these two scores enhances our explanation of the better immune therapy response of the low-risk group and highlights its potential in personalized treatment strategies.

We believe these updates further improve our analysis and provide more biologically meaningful evidence for the immune therapy advantage of the low-risk group. Once again, we appreciate the constructive feedback, and we hope these revisions meet your expectations.

**5. Clinical applicability needs to be discussed, including how this model can guide personalized treatment strategies.**

**Response:** Thank you for your valuable comments on the clinical applicability of our study! Based on your suggestion, we have added content in the Discussion section regarding the clinical applicability of the model and personalized treatment strategies. Specifically, we have discussed in detail how this immune scoring model can assist clinicians in predicting a patient's response to immune therapy based on their immune profile and provide strong support for personalized treatment. Here are the updates in the manuscript:

(1) Clinical applicability of the immune scoring model: We emphasize the potential of the immune scoring model in personalized treatment. By analyzing the TIDE and IPS scores, clinicians can accurately identify which patients are likely to benefit from immune therapy, avoiding ineffective treatment for low-response patients, thereby improving treatment outcomes and reducing side effects. Furthermore, we discuss how treatment strategies can be adjusted based on a patient's risk score. For high-risk patients, combination therapies (e.g., chemotherapy or targeted therapy) may be required, while low-risk patients could benefit from immune checkpoint inhibitors (ICIs) alone.

(2) Personalized treatment decision-making: We further discuss how the model can provide precise prognosis assessments and personalized follow-up management plans for patients. High-risk patients may need more frequent monitoring and proactive interventions, while low-risk patients can focus on long-term follow-up and evaluation after immune therapy. This model allows clinicians to tailor treatment plans for patients, ensuring maximum treatment efficacy and minimizing side effects.

(3) Providing a basis for clinical trial design: We have also added a discussion on how this model can provide a basis for future clinical trial designs by refining inclusion criteria, ensuring the effectiveness and safety of immune therapy, and serving as an efficacy biomarker for evaluating new drugs or treatment regimens.

We believe these added sections provide strong support for the clinical application of the immune scoring model and personalized treatment strategies, further enhancing the practical significance and clinical value of the study. Thank you for your thoughtful suggestions, and we hope these revisions meet your expectations.

**6. Statistical methods must be explicitly stated in every figure legend or relevant text section to ensure clarity and reproducibility.**

**Response:** Thank you for your valuable feedback! We have followed your suggestion and explicitly included the descriptions of the relevant statistical methods in the Methods and Results sections to ensure clarity and reproducibility of the study. Specifically, in the Methods and Results sections, we have detailed the statistical tests used in our analyses, including differential gene analysis, Cox regression analysis, Kaplan-Meier survival curve analysis, and more. We have also described the specific R packages and R version used for each statistical method. With these additions, we believe the manuscript will better ensure reproducibility and transparency. If you have any further suggestions, we will be happy to make further revisions.

Once again, thank you for your careful review, and we believe these revisions will meet your expectations.

**Reviewer #4:**

**Main Issues:**

**1.The proposed ferroptosis-related risk score (nFRGs) demonstrates prognostic value, but its interpretation is fundamentally limited by the absence of treatment data in TCGA/GSE14520. Since HCC outcomes depend heavily on therapies received (e.g., resection, locoregional treatments, systemic therapy), the risk score likely conflates:**

**True prognostic effects (inherent tumor biology).**

**Treatment access/efficacy (e.g., high-risk patients may have been ineligible for curative therapies).**

**This unmeasured confounding biases the model’s hazard ratios and undermines its clinical interpretability.**

**Response:** Thank you for your thorough review of our study! We fully agree with the reviewer’s point regarding the absence of treatment data, which is indeed a significant limitation in our research. Due to the lack of detailed treatment information in the TCGA and GSE14520 databases, our risk score model may be confounded by treatment access and efficacy, as the reviewer pointed out. Specifically, the model might conflate inherent tumor biology with treatment-related factors, such as high-risk patients being ineligible for curative therapies. To address this, we have added the following points to the discussion section of the manuscript:

(1) We emphasize the potential impact of the lack of treatment data on the model and note that high-risk patients may not have received curative treatments or may have received less aggressive therapies, which could affect their prognosis, making it not entirely reflective of tumor biology but rather influenced by treatment effects.

(2) We explicitly point out that unmeasured treatment confounders may bias the hazard ratios of the model, thereby affecting its clinical interpretability.

(3) We suggest that future studies should integrate detailed treatment data, including surgery, chemotherapy, locoregional treatments, and immunotherapy, to better assess the impact of therapy on patient outcomes and optimize existing risk scoring models.

We believe that by incorporating treatment data, future research could further refine the immune scoring model to more accurately reflect the independent effects of tumor biology and treatment, thereby enhancing its clinical usability and interpretability.

Thank you again for your valuable suggestion. We believe these additions will further improve the manuscript and increase its clinical applicability.

**2. Related to the above question: would a sensitivity analysis such as the following be feasible?**

**Stratifying analyses by diagnosis year (as a proxy for treatment era, e.g., pre-/post-sorafenib or immunotherapy availability).**

**Using propensity score matching on clinical covariates (e.g., stage, Child-Pugh class) to approximate balanced treatment access across risk groups.**

Response: Thank you for your valuable suggestions! We fully agree with your focus on treatment factors, particularly the use of stratifying analyses by diagnosis year and applying propensity score matching (PSM) on clinical covariates to control for treatment effects. However, due to the lack of detailed treatment data in the current TCGA and GSE14520 datasets, particularly regarding whether patients received specific treatments (such as sorafenib or immune checkpoint inhibitors), we were unable to perform stratification by treatment era (e.g., pre-/post-sorafenib or immunotherapy) or use PSM with clinical covariates (such as stage, Child-Pugh class) to balance treatment access across risk groups.

We have acknowledged these limitations in the manuscript and suggested that future research could integrate more detailed treatment data, especially information on specific treatments (such as sorafenib or immune checkpoint inhibitors). This would help optimize the risk score model and better evaluate the impact of treatment on prognosis, leading to more accurate treatment strategies and enhancing the clinical applicability and interpretability of the model.

Thank you again for your thoughtful suggestions, and we hope these clarifications address your concerns.

**3.Samples with OS < 30 days or incomplete clinical data were excluded (369 → 341 in TCGA). No imputation methods were described. How do you handle Potential selection bias from complete-case analysis? Have you tried sensitive analysis to evaluate impact of missing data.**

**Response：**Thank you for your insightful comments. In this study, we excluded samples with OS < 30 days to minimize the potential confounding effects of early mortality due to non-tumor-related causes. Additionally, we excluded samples with incomplete clinical data to ensure the reliability of our statistical analyses. We acknowledge that this complete-case analysis approach could introduce selection bias, as patients with missing data were not included in the analysis.

Given the nature and proportion of missing data, we chose not to apply imputation methods, as doing so could introduce additional uncertainty and potential bias that might affect the robustness of the results. Although we did not perform a formal sensitivity analysis, we have implemented strict quality control procedures in the study design phase to minimize the impact of missing data.

We also recognize that this is a limitation, and future studies could explore alternative strategies for handling missing data, such as multiple imputation, to better assess the impact of missing data and selection bias. We have added a discussion of this limitation in the revised manuscript to acknowledge the potential impact of data exclusion on our findings.

**4.Table 1 lists PMIDs for compared models, but some references (e.g., PMID 36290827) are not in the bibliography.**

**Response:** Thank you for your careful review of our manuscript! Regarding the issue raised by the reviewer about PMID 36290827 being listed in Table 1 but not found in the bibliography, we have carefully checked this. We confirm that this reference does exist and is listed in the bibliography as [12]. To avoid any confusion, we will verify the reference section in the revised version to ensure the proper formatting and accurate citation of PMID 36290827. We will ensure that reference [12] is correctly cited. We apologize for any inconvenience caused and appreciate your patience in pointing out this issue. We will make the necessary corrections in the revised manuscript. Thank you again for your valuable feedback.

**5.Median Overall Survival (OS) is not explicitly reported in the manuscript. The Kaplan-Meier curves (Figures 4A–D) show survival differences between high- and low-risk groups but do not provide median OS values for the overall cohort or subgroups.**

Response: Thank you for your thorough review and valuable suggestions! Regarding the point raised by the reviewer about the "absence of explicit reporting of median overall survival (OS)," we have made the necessary changes to the figures. Report of Median OS:We have now explicitly labeled the median overall survival (OS) in the Kaplan-Meier survival curves in Figures 4A–D. In the figure legend, we have added the following note: "Dashed line represents the Median Overall Survival (OS)," to ensure that readers clearly understand that the dashed line represents the median survival time. We believe these changes meet the reviewer’s request and provide clearer figure annotations. Thank you once again for your valuable input.

**6. Number of Events for Primary Endpoint (OS) is not explicitly stated, but calculable from the manuscript. Please provide the median OS and the corresponding number of events in TCGA and GSE14520 cohort.**

**Response:** Thank you for your thorough review and valuable suggestions! Regarding the issue raised by the reviewer about "the number of events for the primary endpoint (OS) not being explicitly stated," we have made the necessary modifications and additions in the revised manuscript. Report of Median OS and Number of Events:

We have now explicitly provided the median OS and the corresponding number of events (survival and death events) for both the TCGA and GSE14520 cohorts in the revised manuscript. Specifically:

In the TCGA cohort: Survival (n=219), Death (n=122), Median OS = 630 days.

In the GSE14520 cohort: Survival (n=136), Death (n=85), Median OS = 1569 days. These data have been reported in detail in the Materials and Methods section of the revised manuscript, allowing readers to clearly understand the number of events and survival times for each group.

Sincerely

Jingjing Huang, M.D.,
