# Supplementary material for: A novel ferroptosis-related signature for predicting prognosis, immune characteristics, and treatment prediction in hepatocellular carcinoma
Source: PLoS One. 2025 Jun 4;20(6):e0322158. doi: 10.1371/journal.pone.0322158 (PMC12136417; doi:10.1371/journal.pone.0322158)
Supplement: Attachment — Submitted filename: Response_to_Reviewers_auresp_3.docx [file pone.0322158.s016.docx]

**Dear Editors and Reviewers：**

We have uploaded all main figure files to the Preflight Analysis and Conversion Engine (PACE) as requested. The PACE-generated files have been successfully downloaded and incorporated into the revised manuscript.

In addition, we have carefully reviewed and adjusted the descriptions of the Supporting Information files to ensure that their sequence and content are fully consistent with the corresponding supplementary materials.

Thank you for your valuable comments regarding the reference list. As requested, we have thoroughly checked all references. During this process, we identified that reference [36] was a duplicate of reference [26]. Accordingly, we have removed reference [36] and renumbered all subsequent references. Both the in-text citations and the reference list have been revised to reflect this correction. We confirm that no retracted articles are included in the revised reference list.

Thank you again for your attention to detail and for your support in improving the quality of our manuscript.

**Reviewer #4:**

**Main Issues:**While I appreciate the authors' acknowledgment regarding the absence of detailed treatment data, I would encourage them to consider a simple stratification by diagnosis year (e.g., pre-/post-2007 for sorafenib approval, or pre-/post-2017 for immune checkpoint inhibitors) as a proxy for treatment era. Even without specific treatment labels, such an analysis could provide additional insights into whether survival differences may partially reflect advances in systemic therapy, and would strengthen the robustness of the prognostic model. I leave this as a suggestion for possible inclusion if feasible.

**Response:**

Thank you for your insightful suggestion regarding stratification by diagnosis year to account for potential differences in treatment era. In our analysis, we did indeed divide the patients into two groups based on their diagnosis year: pre-2007 and post-2007, with 2007 marking a significant shift in the treatment era, especially with the introduction of sorafenib.

Initial survival analysis indicated that the diagnosis year alone was associated with differences in overall survival. However, when patients were further stratified by their risk scores, the survival differences between the pre-2007 and post-2007 groups were no longer statistically significant. This suggests that the diagnosis year does not serve as a decisive factor influencing survival outcomes. Rather, survival disparities are more likely driven by the risk group (high-risk vs low-risk), which further reinforces the stability and robustness of our constructed risk model across different treatment eras.

We would also like to note that the TCGA dataset only includes diagnosis year information up to 2013, which unfortunately makes it impossible to perform stratification based on the introduction of immune checkpoint inhibitors around 2017.

We have included this analysis and clarification in the revised manuscript (Supplementary Material 1, Fig. S3, and Table S2). We believe this additional evaluation further validates the robustness of the prognostic model. Thank you again for your valuable suggestion.

Sincerely

Jingjing Huang, M.D.,
